# Asialo-rhuEPO as a Potential Neuroprotectant for Ischemic Stroke Treatment

**DOI:** 10.3390/ph16040610

**Published:** 2023-04-18

**Authors:** Farooqahmed S. Kittur, Chiu-Yueh Hung, P. Andy Li, David C. Sane, Jiahua Xie

**Affiliations:** 1 Department of Pharmaceutical Sciences, Biomanufacturing Research Institute & Technology Enterprise, North Carolina Central University, Durham, NC 27707, USA; chiuyueh@gmail.com (C.-Y.H.); pli@nccu.edu (P.A.L.); 2 Carilion Clinic and Virginia Tech Carilion School of Medicine, Roanoke, VA 24014, USA; dcsane@carilionclinic.org

**Keywords:** multimodal neuroprotectant, erythropoietin, hematopoietic activity, asialo-erythropoietin, non-erythropoiesis, erythropoietin receptor, cerebral ischemia and reperfusion, preclinical study, clinical trial

## Abstract

Neuroprotective drugs to protect the brain against cerebral ischemia and reperfusion (I/R) injury are urgently needed. Mammalian cell-produced recombinant human erythropoietin (rhuEPO^M^) has been demonstrated to have excellent neuroprotective functions in preclinical studies, but its neuroprotective properties could not be consistently translated in clinical trials. The clinical failure of rhuEPO^M^ was thought to be mainly due to its erythropoietic activity-associated side effects. To exploit its tissue-protective property, various EPO derivatives with tissue-protective function only have been developed. Among them, asialo-rhuEPO, lacking terminal sialic acid residues, was shown to be neuroprotective but non-erythropoietic. Asialo-rhuEPO can be prepared by enzymatic removal of sialic acid residues from rhuEPO^M^ (asialo-rhuEPO^E^) or by expressing human *EPO* gene in glycoengineered transgenic plants (asialo-rhuEPO^P^). Both types of asialo-rhuEPO, like rhuEPO^M^, displayed excellent neuroprotective effects by regulating multiple cellular pathways in cerebral I/R animal models. In this review, we describe the structure and properties of EPO and asialo-rhuEPO, summarize the progress on neuroprotective studies of asialo-rhuEPO and rhuEPO^M^, discuss potential reasons for the clinical failure of rhuEPO^M^ with acute ischemic stroke patients, and advocate future studies needed to develop asialo-rhuEPO as a multimodal neuroprotectant for ischemic stroke treatment.

## 1. Introduction

Stroke remains a major cause of mortality and long-term disability globally. Although the age-standardized rates of incidence and death from strokes have decreased since 1990, the annual number of deaths due to stroke increased substantially, with over 12 million incidents and more than 100 million prevalent cases worldwide [1]. Consequently, stroke and post-stroke care continue to present major social and economic challenges for society. Strokes are mainly classified as either ischemic or hemorrhagic, with the former accounting for approximately 87% of cases [1,2]. Following an ischemic stroke (IS) caused by the blockage of an artery in the brain, a lack of cerebral blood supply immediately causes severe oxygen and glucose deprivation in the blocked region, leading to suppression of ATP production, reduction of pH, impairment of mitochondrial dynamics, inhibition of glycosylation capacity, and increased production of reactive oxygen species (ROS) [3,4,5,6,7]. These pathophysiological changes result in neuronal cell injury and death [2,3]. Timely reperfusion by thrombolysis or mechanical thrombectomy to re-establish blood flow is an effective therapeutic strategy to salvage damaged nerve cells and improve clinical outcomes by reducing cerebral ischemic damage and preserving brain functions [2,3,6]. However, reperfusion paradoxically exacerbates brain tissue injury [3,8,9]. Therefore, adjunct neuroprotection alongside reperfusion is crucial and may bring better outcomes.

Cerebral ischemia and reperfusion (I/R) injury is a detrimental process, since both I/R processes can cause various pathophysiological changes. The damaging effects induced by ischemia, reperfusion, or their combined action are not separable [3,8,10]. The mechanisms underlying cerebral I/R injury are complex and include metabolism dysfunction, mitochondrial dysregulation, oxidative stress, disruption of blood–brain barrier (BBB), leukocyte infiltration, brain inflammation, and various types of cell death, such as necrosis, apoptosis, autophagy, necroptosis, and pyroptosis [3,9,11,12]. While the detailed mechanisms are not fully understood, these complex pathophysiological changes ultimately lead to infarction and subsequent cognitive impairments [3,10,11]. Nevertheless, the discovery of neuroprotective therapeutics and further understanding the complexity of the I/R-induced injury cascade are crucial for developing improved treatments.

Based on the current understanding of the I/R injury, various pharmacological and mechanical interventions have been explored to protect the brain from its detrimental effects with some efficacy. These interventions include inhibiting apoptosis, promoting angiogenesis, suppressing the immune system and inflammation, reducing ROS production and stress response, and regulating metabolic processes [2,3,7,10,13]. Unfortunately, most of these strategies failed in clinical trials, leaving no safe and effective therapeutic treatment to ameliorate the repercussions of cerebral I/R injury [2,10,13]. Moreover, therapies targeting a single specific mechanism have been found to be insufficient, as I/R injury involves multiple pathophysiological pathways [14,15]. Thus, an agent targeting an array of key cellular pathways has been proposed in order to have better long-term benefits [14,15,16].

Erythropoietin (EPO) is a glycoprotein hormone primarily known for regulating red blood cell (RBC) production (Table 1) [17]. In addition to its hematopoietic activity, EPO and its derivatives have been demonstrated to display remarkable anti-apoptotic and broad tissue-protective effects against damage triggered by I/R injury, or cytotoxic agents in the brain, the heart, the kidneys, and the liver [18,19,20]. EPO and the EPO receptor (EPOR) are functionally expressed in various non-hematopoietic organs [18,21,22], including neurons, glial cells, and endothelial cells in the central nervous system (CNS) [23,24]. Recombinant human EPO produced in mammalian cells (rhuEPO^M^) has been demonstrated to display remarkable neuroprotection in animal models of ischemic stroke [25,26,27]. Most importantly, numerous studies have also revealed that its tissue-protective properties are mediated through pleiotropic effects, including anti-oxidative, -apoptotic, -inflammatory, and -excitotoxic effects, as well as angiogenic and neurogenic effects [27,28,29,30].

**Figure 1:**
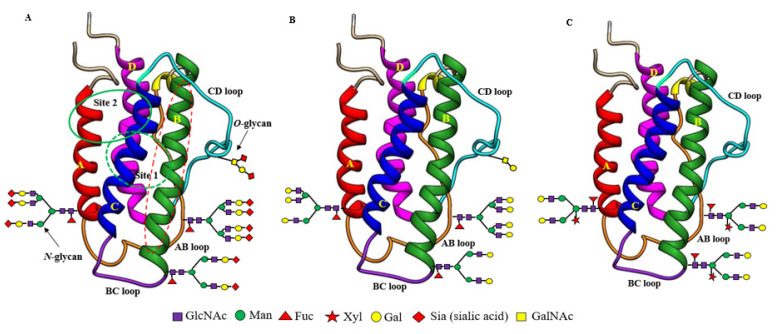
Structural differences between recombinant sialylated rhuEPO^M^ (**A**), enzymatically prepared asialo-rhuEPO^E^ (**B**) and plant-produced asialo-rhuEPO^P^ (**C**). Their protein structures are the same with two separate hematopoietic receptor (EPOR)_2_ binding sites (site 1 marked by the green dotted circle at the back side of EPO; site 2 marked by a solid green circle at the front side of EPO). Both binding sites are present distal to the carbohydrate chains. Helix B marked by the red dotted oval is proposed to be involved in EPO-mediated protective functions. All three types of EPO differ with respect to the structure of their *N*-glycan chains. RhuEPO^M^ bears sialic acid residues (red diamond) as terminal sugar on bi-, tri- and tetrantennary *N*-glycans while asialo-rhuEPO^E^ lacks sialic acid residues and contains β1,4-galactose residues (yellow circles) as terminal sugars. Asialo-rhuEPO^P^ lacks terminal sialic acid residues like asialo-rhuEPO^E^. In addition, asialo-rhuEPO^P^ bears biantennary *N*-glycan chains with plant-specific xylose and fucose residues and lacks *O*-glycan chain.

Given the broad tissue-protective and pleiotropic effects, rhuEPO^M^ entered into clinical trials with acute IS patients two decades ago [31], which was followed by several other trials [32,33,34]. Unfortunately, its tissue-protective effects to reduce the infarct size from cerebral I/R injury could not be consistently observed in clinical studies [32]. Its hematopoietic activity-associated side effects (HAASEs), such as hypertension and thrombosis, were believed to mask tissue-protective effects. As a result, scientists developed low- or non-erythropoietic tissue-protective derivatives, such as EPOL (EPO derived from genetically modified goat milk with low hematopoietic activity), Neuro-EPO (EPO produced from mammalian cell with low sialic acid content), CEPO (carbamoylated EPO), asialo-rhuEPO (EPO lacking terminal sialic acid) (Figure 1), and MEPO (mutant EPO made by replacing a single amino acid within the erythropoietic motif) and other EPO variants, conceptually devoid of side effects for brain protection as reviewed by Ma et al. [6]. Among them, asialo-rhuEPO was well-documented to be nonerythropoietic but neuroprotective [35,36,37,38,39,40,41]. It has also been demonstrated to have protective effects on other organs (e.g., heart) from I/R injury [42,43,44].

This review summarizes recent progress on neuroprotection studies performed with enzymatically prepared asialo-rhuEPO (asialo-rhuEPO^E^) and plant-produced asialo-rhuEPO (asialo-rhuEPO^P^) as well as rhuEPO^M^ (Table 1) on the cerebral I/R injury. It also discusses potential factors responsible for rhuEPO^M^’s clinical failures and the crucial studies needed on asialo-rhuEPO to warrant its success in future clinical trials.

## 2. EPO and RhuEPO^M^

### 2.1. EPO Structures and Properties

As mentioned earlier, EPO (Table 1) is a glycoprotein secreted primarily by the kidneys in adults [45], with small amounts secreted by the liver [46] and the brain [47]. EPO protein encoded by the *EPO* gene is 193 amino acids long with a 27 amino acid long *N*-terminal signal peptide. Shortly before secretion, the signal peptide is cleaved, leaving behind matured EPO with only 166 amino acids. However, the physiologically active form circulating in the plasma is 165 amino acids long because of the removal of *C*-terminal Arg^166^ by proteolysis [48]. The active mature EPO contains two intramolecular disulfide bonds (Cys7-Cys161, Cys29-Cys33) and bears three *N*-glycan chains attached to Asn at positions 24, 38, and 83 and an *O*-glycan chain at Ser 126 [49]. Therefore, EPO is a heavily glycosylated protein with ~40% carbohydrate content and a molecular weight of ~30,400 Daltons [50]. The three-dimensional structure determined by NMR [51] and X-ray crystallography [52] showed that EPO is an elongated molecule consisting of a left-handed, four-helix bundle (Figure 1A), typically present in the hematopoietic growth factor family members. The four long helices (A, B, C, and D) are arranged in an up-up-down-down direction, linked by two long cross-over loops (AB and CD) and one short loop (BC) (Figure 1A). Two distinct patches of amino acids on the protein surface were shown to form two spatially separate binding sites for its homodimeric hematopoietic receptor (EPOR)_2_: a high-affinity binding site (Site 1, dotted green circle at the back side of EPO), comprising helix D and the AB loop, and a low-affinity binding site (Site 2, solid green circle at the front side of EPO) in the A and C helical bundle (Figure 1A). EPO thus forms a 2:1 homodimeric (EPOR)_2_:EPO complex.

**Figure 2:**
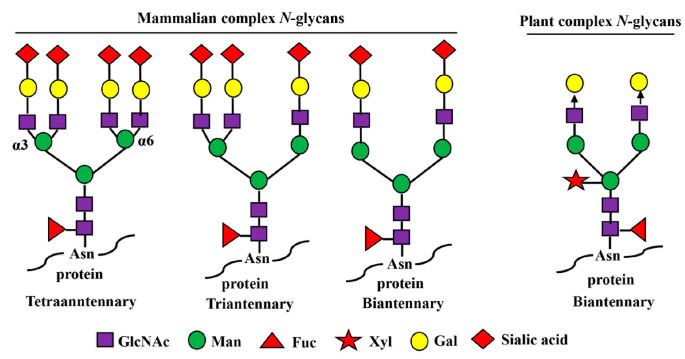
Mammalian- and plant-produced complex-type *N*-glycans. Complex *N*-glycans refer to those in which both the α3- and α6-linked mannose residues are substituted with GlcNAc moieties. In mammals, the *N*-glycan chains can be bi-, tri-, and tetraantennary, and the GlcNAc residues in each glycan chain are further extended with β1,4-galactose residues and terminal sialic acid residues. The sialic acid in humans is *N*-acetyl-neuraminic acid (Neu5Ac) while other mammals have both Neu5Ac and *N*-glycolylneuraminic acids (GlcNGc). In plants, their complex *N*-glycan chains are biantennary, but do not bear β1,4-galactose and sialic acid residues. The arrow depicts the locations of further expansion of plant complex *N*-glycans with β1,4-galactose residues by overexpression of human β1,4-galactosyltransferase gene (*GalT*).

The carbohydrate (*N*-glycan) chains in EPO are clustered at one end of the molecule, distal from the receptor-binding sites (Figure 1A). Although the carbohydrate chains constitute approximately 40% of the mass of EPO, they are thought to cover much of the surface of the molecule, but not to be involved in (EPOR)_2_ binding even though they have been shown to influence EPO’s in vivo hematopoietic activity [53]. The smaller *O*-glycan chain at Ser 126 has not been found to have any important roles in both in vitro and in vivo hematopoietic activity of EPO yet [53,54] while the *N*-glycan chains are proven to be indispensable for its secretion, proper folding, stability, and in vivo hematopoietic activity [53,55,56]. Each *N*-glycan chain in EPO is branched containing 2 to 4 arms (or antennas) capped with negatively charged sugar residue, sialic acid (*N*-acetylneuraminic acid, Neu5Ac) (Figure 1 and Figure 2), which imparts a net negative charge on the protein molecule, giving EPO an acidic pI in the range of 3.92 to 5.11 (Table 1) [57]. Theoretically, EPO can have around 14 sialic acid residues, with 12 present at 3 *N*-glycan chains and 2 presenting at 1 *O*-glycan chain.

Currently, transfected Chinese hamster ovary cells (CHOs) are the major source for large-scale manufacturing of rhuEPO^M^ (Table 1) [50]. Both plasma EPO and rhuEPO^M^ have been reported to exist in numerous glycoforms due to the heterogeneity of their *N*- and *O*-glycan chains and varying sialic acid content [58]. The former was reported to bear only mono-, di-, and triantennary *N*-glycans but lack tetraantennary *N*-glycan chains while the latter was shown to contain predominantly tetrantennary sialylated *N*-glycans [58]. The degree of branching has been shown to influence its in vivo hematopoietic activity, and rhuEPO^M^ bearing tetraantennary *N*-glycans displays higher activity than that with biantennary *N*-glycans [59]. The terminal sialic acid residues on glycan chains were reported to play a critical role in the in vivo hematopoietic activity of EPO by prolonging its serum half-life (~4–8 h) [60,61]. Removal of sialic acid residues was shown to result in an almost complete loss of its in vivo hematopoietic activity, while its in vitro activity remains unaffected [62,63]. The near complete loss of in vivo hematopoietic activity could be due to the rapid clearance of EPO from the circulation system (short half-life, ~2–3 min) [35,64] likely through asialoglycoprotein receptors present on the liver [62,63].

Concerning the influence of carbohydrate chains on the tissue-protective activity of EPO and its derivatives, the neuroprotection studies from both Yamashita et al. [39] and our own [41] suggest that they do not have any influence on the short-term protective effects because both rhuEPO^M^ and asialo-rhuEPO^P^ displayed similar protection in in vivo model of transient I/R injury. However, there is no report of their impact on long-term outcomes of EPO-mediated neuroprotection. Concerning EPO protein regions involved in binding to receptors to transduce tissue-protective signals, both binding sites 1 and 2 that are essential for erythropoiesis were shown to be not required for tissue-protective function, because chemical and mutational modifications of amino acid residues within these sites did not abolish the protective function of EPO and its derivatives [65,66]. Brines et al. [67] proposed that the region (Gln^58^-Ser^82^) comprising the B helix in EPO (see Figure 1A,B helix colored green with dotted red oval) is sufficient for its tissue-protective function. Thus, the regions of EPO responsible for its hematopoietic and tissue-protective functions are likely different.

### 2.2. Neuroprotective Function of RhuEPO^M^ in Preclinical Studies

EPO was originally thought to function as the regulator of erythropoiesis only. However, in 1987 it was recognized that rhuEPO^M^ possesses cognition-enhancing properties when given to anemia patients suffering from kidney failure or under chemotherapy [68]. Research performed in the following decade showed that EPO and EPOR are in fact widely distributed in the mammalian brain [69,70], and are up-regulated after ischemic infarction or hypoxic damage [71]. Targeted deletion of EPO and EPOR in mice showed, in addition to defective erythropoiesis, increased apoptosis in the mouse brain, suggesting that EPO signaling is required for brain development [72,73]. The above observations led to the hypothesis that both endogenously induced EPO and exogenously added rhuEPO^M^ could provide neuroprotection.

In a series of elegant experiments, the Sasaki group [25,74] showed that exogenous rhuEPO^M^ infused directly into the brain resulted in strong protection against ischemic injury, whereas soluble EPOR infused into the brain worsened the injury by neutralization of endogenous EPO. In subsequent studies, intracerebral injection of rhuEPO^M^ before permanent occlusion of the middle cerebral artery (MCA) in mice revealed a 50% reduction of infarct volume [75], whereas Brines et al. [18] using a rodent model of cerebral ischemia where MCA was transiently occluded, showed that systemic administration of rhuEPO^M^ before or up to 6 h after ischemia reduced injury by 50–75%. Intraperitoneal administration of rhuEPO^M^ (5000 IU/kg bw) in rats was shown to reduce the ischemic area in the cerebrum, cerebellum, and brain stem [76], while Villa et al. [77] reported similar doses of exogenous rhuEPO^M^ reducing astrocyte activation and the recruitment of leukocytes and microglia into an infarction produced by MCA occlusion (MCAO) in rats and decreasing the production of inflammatory cytokines. Gunnarson et al. [78] reported that EPO decreased the susceptibility of the brain to edema via regulation of brain-water homeostasis. We have also observed that intravenous (IV) administration of rhuEPO^M^ (44 µg/kg bw; EPO: ~8 ng/IU) in mice at the restoration of blood flow following MCAO reduced the infarct volume and brain edema by 50% [41]. In other animal studies involving observation of functional outcomes in addition to infarct volume, rhuEPO^M^ was shown to improve learning ability [74], navigation disability [25], sensory neglect, and sensorimotor functions [79,80]. RhuEPO^M^ was also reported to be neuroprotective in animal models of hemorrhagic stroke [81] and traumatic brain injury (TBI) [82,83]. All of the above-discussed studies, together with many others not mentioned in this article, demonstrated that EPO is neuroprotective and could possibly be used to treat brain injury.

### 2.3. Neuroprotection Mechanisms of EPO

Following the discovery of its excellent neuroprotective effects, the mechanisms of EPO-mediated neuroprotection were also partly elucidated. Numerous studies have shown that it protects the CNS by limiting the production of ROS and glutamate, reducing BBB disruption, modulating inflammation, balancing the mitochondrial fission–fusion dynamics, attenuating apoptosis, stimulating angiogenesis, and inducing neurogenesis. RhuEPO^M^ was shown to block the generation of ROS [84] and preserve the BBB integrity [85,86] and the cellular integrity in neurons [87] and inflammatory cells of the nervous system [88]. It has been reported to protect neurons from glutamate toxicity either by repressing Ca^2+^ influx [89] or through the suppression of Ca^2+^/calmodulin-dependent neuronal nitric oxide synthase (nNOS) [25]. The neuroprotective effects of EPO are now believed mainly due to its anti-apoptotic properties, akin to that observed in erythropoiesis. In the case of neurons, its protective effects were shown to occur through the prevention of mitochondrial depolarization via control of BAD, BAX, and PUMA, and the balance of mitochondrial dynamics through restoration of I/R injury-dysregulated mitochondrial fission and fusion proteins, leading to prevention of cytochrome *c* (cyt *c*) release and activation of caspases [41,87,90,91,92]. Moreover, rhuEPO^M^ treatment was found to upregulate the anti-apoptotic proteins X-linked inhibitor of apoptosis (XIAP) and inhibitor of apoptosis proteins (c-IAP2) [93]. RhuEPO^M^ was also reported to be anti-inflammatory, and has been shown to reduce the infarct volume in an animal model of cerebral ischemia by decreasing the production of pro-inflammatory molecules like TNF, IL-6, and chemokine MCP-1 [77]. The anti-inflammatory effect of EPO is reported to be due to the inhibition of apoptosis, as well as the reduction of BBB disruption to regulate immune-cell trafficking across the BBB, and attenuation of microglia activation [6,86,94].

Besides direct effects on neurons, EPO-mediated neuroprotection is believed to be due to improvement in cerebral blood flow (CBF) and brain perfusion by promoting new vessel growth. Beleslin-Cokic et al. [95] showed that EPO can stimulate endothelial nitric oxide synthase (eNOS) and nitric oxide (NO) production, which could improve CBF and contribute to neuroprotection. Furthermore, Cruz-Navarro et al. [96], using wild-type and eNOS-deficient mice, reported that EPO-mediated improvement in CBF in a traumatic brain injury model is eNOS-dependent. The angiogenic effect of rhuEPO^M^ was also confirmed in mice genetically engineered to lack EPO and EPOR, whose embryos displayed severe defects in angiogenesis [97], while rhuEPO^M^ treatment could promote the angiogenesis through the activation of AMPK-KLF2 signaling pathways [98]. EPO also exhibits neurotrophic function. It was shown to promote the survival and differentiation of dopaminergic precursor neurons [99]. In addition, endogenously produced EPO under hypoxia was reported to act directly on neuronal stem cells in the forebrain, suggesting a direct role in neurogenesis following hypoxia [100]. In sum, these studies revealed that EPO-mediated neuroprotection involves multiple mechanisms.

**Figure 3:**
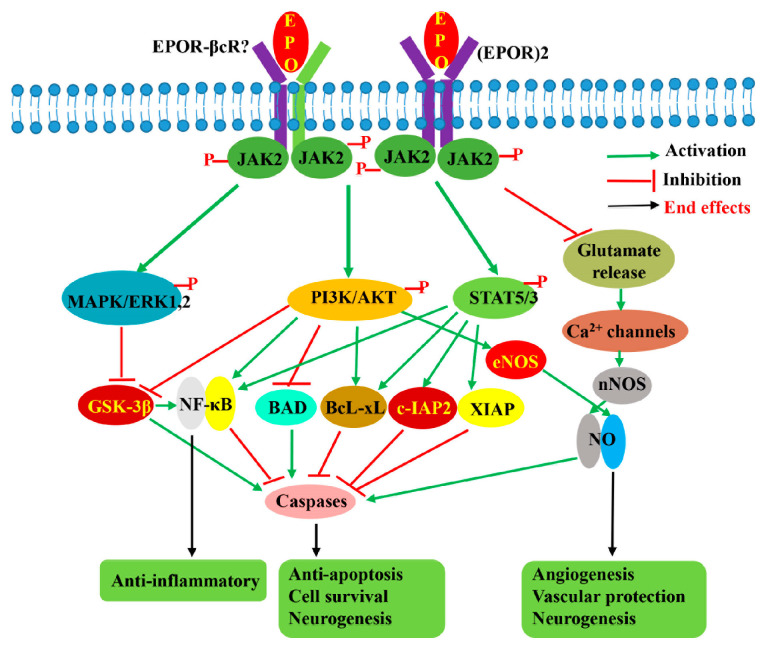
EPO-induced signaling pathways involved in its neuroprotective effects. EPO by binding to either homodimeric (EPOR)_2_ receptor or another possible heterodimeric receptor EPOR-βcR activates JAK2 by phosphorylation, followed by activation of downstream STAT5, PI3K/AKT, and MAPK signaling pathways, as well as regulation of voltage-gated Ca^2+^ channel. The activation of these three signaling pathways results in the activation of anti-apoptotic and anti-inflammatory pathways benefiting cell survival while promoting angiogenesis and neurogenesis. The suppression of glutamate release benefits cell survival and inhibits apoptosis through the regulation of voltage-gated calcium ion channels to lower glutamate excitotoxicity.

Concerning the signaling pathway involved in EPO-mediated neuroprotection, many studies have shown that multiple signaling pathways, which differ in importance depending on the cell type, type of injury, and EPO administration with respect to the injury, are activated by EPO. Like erythropoiesis, neuroprotective signals are reported to be initiated by phosphorylation of JAK2, followed by activation of downstream STAT5, PI3K/AKT, and MAPK/ERK1/2 pathways [71,101,102,103,104] (Figure 3). Siren et al. [71] reported that in hippocampal neurons, rhuEPO^M^ restored the levels of phosphorylated STAT5, AKT, and ERK1/2 reduced by hypoxia, suggesting that these three pathways are important for EPO-mediated neuroprotection. In another study using EPOR mutants in which a component of the downstream signaling pathway was either selectively retained or inhibited [103], it was revealed that STAT5 and PI3K/AKT pathways in the SH-SY5Y cell line are critical because their impairments completely abolished the anti-apoptotic effects of rhuEPO^M^, whereas the MAPK pathway is less important. In addition, the authors suggested that both AKT and STAT5 also contribute to the activation of the NF-κB pathway [103]. Digicaylioglu and Lipton [93] also reported that JAK2 phosphorylated NF-κB’s inhibitory subunit, IκB, releasing the transcription factor NF-κB, and allowing its translocation to the nucleus to activate the expression of neuroprotective genes. In an in vivo study, Kilic et al. [102] also demonstrated that dual activation of ERK-1/2 and Akt is crucial for EPO’s neuroprotective activity. STAT5 activation was shown to inhibit apoptosis by inducing the synthesis of the protein Bcl-xL [105], whereas activation of ERK1/2 and AKT was reported to induce Bcl-xL, inactivate pro-apoptotic BCL2-associated agonist of cell death (BAD), and glycogen synthase kinase 3β (GSK3B), resulting in inhibition of apoptosis [88,102]. In addition to mediating anti-apoptotic effects, STAT5, PI3K/AKT, and MAPK pathways are also thought to be responsible for the antioxidant, anti-inflammation, angiogenesis, and neurogenic effects of EPO. Besides these three pathways, EPO was reported to display its neuroprotective effects through stimulation of eNOS [95] and activation of voltage-gated Ca^2+^ channels, resulting in NO production [95,106], as well as via regulation of the Wnt1 signaling pathway to prevent microglial early- and late-stage apoptotic injury [88].

### 2.4. Clinical Trials of RhuEPO^M^ in Acute IS Treatment

After preclinical studies demonstrated its broad tissue-protective properties and pleiotropic effects, rhuEPO^M^ entered clinical trials two decades ago to investigate its neuroprotection against I/R injury [31]. Although several clinical trials were conducted in different locations, unlike preclinical studies, its neuroprotective effects could not be consistently reproduced in clinical studies [31,32,33,34] (Table 2). In the earliest pilot study with 40 acute IS patients, daily IV administration of 33,000 IU rhuEPO^M^ for the first three days, with the first injection within eight hours of the onset of stroke symptoms, showed a significant reduction in infarct size and neurological deficit, recovery of neurocognitive function, and amelioration of stroke-related disability at 30 days [31]. The observed positive effects of rhuEPO^M^ were supported by the other two subsequent small clinical trials [33,34]. In a randomized trial involving 37 patients in the rhuEPO^M^ group and 43 in the control group, a high dose of 56,000 IU (initial IV administration of 16,000 IU, followed by 8000 IU every 12 h over 60 h) was found to be effective in improving neurological function at 14 and 28 days, respectively [33]. Even a low-dose trial with 71 acute IS patients showed improvement in long-term (five years) neurological outcomes and a lower 90-day recurrent stroke rate with two consecutive subcutaneous (SC) administrations of 5000 IU rhuEPO^M^ each at 48 and 72 h after onset of ischemic stroke symptoms [34]. The same group also demonstrated that the improvement in recurrent stroke rate with a low dose of rhuEPO^M^ was through the enhancement of circulating endothelial progenitor cell levels [107]. Unfortunately, a large trial involving 522 acute IS patients with a cumulative dose of 120,000 IU (three doses with 40,000 IU each IV injection at 6, 24, and 48 h after onset of stroke symptoms) did not show any beneficial effects and even displayed harmful outcomes with increased mortality, particularly when combined with rtPA treatment [32].

### 2.5. Possible Factors Responsible for Clinical Failure of RhuEPO^M^ in Ischemic Stroke Treatment

After the clinical trials of rhuEPO^M^ against cerebral I/R injury proved unsuccessful, concerns were raised regarding its capacity to cross the BBB due to its large molecular weight of approximately 30.4 kDa [50]. However, several research groups [18,108,109,110] have reported that rhuEPO^M^ can cross the BBB either through EPOR-mediated or non-receptor-mediated transport, indicating that BBB permeability may not be an issue. During cerebral I/R injury, the BBB’s integrity is interrupted, making it easier for injected rhuEPO^M^ to cross the barrier [111]. In addition, rhuEPO^M^ therapy for cardiac and renal protection also showed no clear efficacy in clinical studies, even though animal data had shown outstanding protective effects against I/R injury in the heart [19,112,113] and kidneys [22]. Several systematic reviews and meta-analyses concluded that the observed cardioprotective effects of rhuEPO^M^ to reduce MI size in animal models of myocardial I/R injury could not be reliably translated in clinical settings [114,115,116,117]. Taken together, these findings suggest that the impermeability of the BBB to rhuEPO^M^ is unlikely to be the major reason for its failure in stroke clinical trials, and thus other factors must be responsible.

Besides the issue of BBB permeability, several other factors were also considered responsible for the clinical failures of rhuEPO^M^ in stroke treatment. These include the dosage, the age of the patients, and most critically, its HAASEs, such as hypertension and thrombosis [26,30,117,118,119]. Regarding HAASEs with rhuEPO^M^ administration, studies in transgenic mice have demonstrated that high levels of EPO induced by overexpressing the *EPO* gene can increase hematocrit, leading to vasoconstriction and cardiac dysfunction [120,121]. Similarly, in humans, rhuEPO^M^ treatment for anemia patients with a dose of 100 IU/kg bw has been found to have pro-thrombotic or platelet-activating effects and cause hypertension in 20–30% of renal patients [122,123]. Since rhuEPO^M^ doses for tissue-protection purposes are at least five times higher than those required for anemia treatment [29], these higher doses can stimulate mass production of RBCs and increase the risk of thrombosis. Therefore, the HAASEs associated with rhuEPO^M^ could be a major issue when used for tissue protection.

The HAASEs of rhuEPO^M^ were considered in previous clinical trials. In order to minimize side effects, lower doses of rhuEPO^M^ were used compared to preclinical studies. However, these lower doses likely pose two new problems: insufficient dose and a narrow therapeutic window, hindering the display of its tissue-protective effects. Preclinical studies on neuroprotection have used doses of rhuEPO^M^ ranging from ~1000 to ~5950 IU/kg bw, with 5000 IU/kg being the most common [6,124]. In contrast, clinical trials have used rhuEPO^M^ doses of ~150 to ~1850 IU/kg bw (assuming an average patient weight of 65 kg) [31,32,33,34]. The clinical doses are several-fold lower than those used in preclinical studies. In cardioprotection clinical trials, lower doses of rhuEPO^M^ [114,117] were also used compared to preclinical studies [119,125,126]. Low doses of rhuEPO^M^ used in clinical trials in general showed no consistent benefits against I/R injury in neuroprotection, cardioprotection, and renal protection, raising concerns about the dosage applied [119,124,127]. Moreover, it is still not clear whether rhuEPO^M^ uses the classical (EPOR)_2_ or the alternative tissue-protective receptors, such as EPOR-beta common receptor (βcR) and ephrin B4 (EphB4), to display its tissue-protection function. The alternative tissue-protective receptors require much higher concentrations of rhuEPO^M^ than for erythropoiesis [29,127], because both EPOR-βcR [128] and EphB4 [129] were found to have very low affinity and may require a high concentration of rhuEPO^M^ to transduce a tissue-protective signal. Additionally, the administration times of rhuEPO^M^ in clinical studies are generally much later than those in preclinical studies, which may also be a responsible factor in the clinical failure of the drug. Currently, EPO’s therapeutic window for brain protection is not well-established, and may depend on the type of brain injury and the dosage and route of administration. Extending the therapeutic time window could allow rhuEPO^M^ to display its protective effects. In one animal study, high doses of rhuEPO^M^ for cardioprotection could extend the therapeutic time window [125].

The above discussion clearly indicates that in order to bring about stable and beneficial effects, as well as to extend the treatment time window, high doses of rhuEPO^M^ should be used in clinical trials. Unfortunately, high doses of rhuEPO^M^ have been found to correlate with severe HAASEs [30,119,124]. High doses may cause untoward complications, e.g., polycythemia and thrombotic secondary stroke [130]. Some studies have reported increased rates of adverse cardiovascular events with high doses of rhuEPO^M^ [119,131]. Therefore, its HAASEs limited the application of high rhuEPO^M^ dosage in clinical trials. A promising approach to increase rhuEPO^M^ doses and prolong the therapeutic window could be to use nonerythropoietic EPO derivatives, such as asialo-rhuEPO to avoid the HAASEs.

## 3. Asialo-rhuEPO

### 3.1. Asialo-rhuEPO Structures and Properties

EPO lacking terminal sialic acid residues is known as asialo-rhuEPO, which contains neutral galactose residues as new terminal sugars instead of acidic sialic acid residues (Table 1; Figure 1B). Removal of sialic acid residues does not impact protein folding [56], but has shown to dramatically alter protein charge, binding capacity to the homodimeric (EPOR)_2_, in vitro and in vivo hematopoietic activities [60,61], and circulatory half-life [17,35]. In contrast to sialylated EPO with acidic pI, asialo-rhuEPO is a basic protein with a pI of 8.5 and a short circulatory half-life of ~2.5 min [35]. It has been reported to bind to the EPOR four times faster than its sialylated form in vitro [132]. Most importantly, asialo-rhuEPO was demonstrated to lack in vivo hematopoietic activity (non-erythropoietic) even at very high doses [35], and could cross the BBB to display excellent neuroprotective effects [35,36,38,39,41,133].

### 3.2. Methods of Asialo-rhuEPO Production

Currently, there are two methods used to produce asialo-rhuEPO (Table 1). It is commonly prepared by enzymatic removal of sialic acid residues (desialylation) from rhuEPO^M^ [35,56]. We designate this enzymatically prepared one as asialo-rhuEPO^E^. Recently, plants have been successfully glycoengineered to produce asialo-rhuEPO [134,135,136,137], which is designated as asialo-rhuEPO^P^ in this review.

#### 3.2.1. Enzymatic Method

Asialo-rhuEPO in small quantities for basic research was produced by the enzymatic method. In this method, asialo-rhuEPO^E^ was prepared by desialylation of rhuEPO^M^ with commercially available enzymes called neuraminidases (also known as sialidases) [35,56,132]. Neuraminidases catalyze the hydrolysis of α2,3-, α2-6-, α2-8-, and α2-9-linked Neu5Ac (a type of sialic acid typically present on mammalian glycoproteins) from glycoproteins. Although this is a simple and straightforward method to obtain asialo-rhuEPO without the loss of biological activity, it is not an economically viable method for large-scale production because of the high cost (~4000 USD/mg protein) of rhuEPO^M^ [138]. In addition, neuraminidases for the above purpose are unavailable in bulk quantities for large-scale desialylation of rhuEPO^M^. A mammalian cell-based expression system is also not available to directly express asialo-rhuEPO. Hence, the neuroprotective properties of asialo-rhuEPO^E^ could not be translated into clinical practice. Alternative methods to produce asialo-rhuEPO inexpensively were therefore sought to realize its full therapeutic potential.

#### 3.2.2. Plant-Based Expression Method

Plants have been used as an inexpensive expression system to produce asialo-rhuEPO because they lack sialylation capacity (as they lack the entire enzymatic machinery necessary for the synthesis and transfer of sialic acid residues to glycoproteins) but have the ability to synthesize similar complex biantennary *N*-glycans like mammalian cells (Figure 2) [139,140,141]. Moreover, transgenic plants expressing wild-type or chimeric human *GalT* can sufficiently add galactose residues on the *N*-glycans of produced glycoproteins (Figure 2) [142,143]. The other advantages of using a plant-based expression system are low production cost, lack of human pathogen contamination, and ease of scaling up in production [140,144]. We produced asialo-rhuEPO in tobacco plants by stably co-expressing human *EPO* and β1,4-galactosyltransferase (*GalT*) genes [135,136,137], while Parson and co-workers produced it in moss [134].

### 3.3. Unique Properties of Asialo-rhuEPO^P^

#### 3.3.1. Asialo-rhuEPO^P^ Carries Plant-Specific Biantennary *N*-Glycans

Asialo-rhuEPO^P^ accumulates as 28–30 kD bands representing different glycoforms in transgenic tobacco plants [136]. It is 162 amino acids long because of the proteolytic removal of the extreme *C*-terminal region Thr^163^-Arg^166^ [145]. This lost region is not important for the biological activity of EPO [146]. Asialo-rhuEPO^P^ is a basic protein with a theoretical pI of 8.75. All three *N*-glycosylation sites in asialo-rhuEPO^P^ are occupied with *N*-glycan chains each bearing terminal mammalian-type β1,4-galactose residues [136,137]. The proportion of β1,4-galactose residues on asialo-rhuEPO^P^ was high (72%) when chimeric *GalT* was co-expressed with *EPO* [137], whereas it was low (8%) in the case of co-expressing wild-type *GalT* [136]. The *N*-glycan chains in asialo-rhuEPO^P^ are slightly different from that in asialo-rhuEPO^E^. Asialo-rhuEPO^P^ carries biantennary *N*-glycans containing plant-specific β1,2-xylose and α1,3-fucose residues, while those *N*-glycans in asialo-rhuEPO^E^ lack β1,2-xylose but contain a core α1,6-fucose instead of α1,3-fucose (see Figure 2). The presence of biantennary *N*-glycans with plant-specific β1,2-xylose and α1,3-fucose residues on asialo-rhuEPO^P^ showed no impact on in vitro (EPOR)_2_ binding, since asialo-rhuEPO^P^ displayed similar affinity for the (EPOR)_2_ as rhuEPO^M^ [135]. Furthermore, it provided a similar level of protection to the brain as rhuEPO^M^ after I/R injury [41], suggesting that the biantennary *N*-glycans bearing plant-specific sugars have no impact on its in vivo neuroprotective activity.

#### 3.3.2. Asialo-rhuEPO^P^ Is Non-Erythropoietic and Non-Immunogenic

RhuEPO^M^ doses used for neuroprotection are typically higher (~40 µg/kg bw in a rodent stroke model and 4–36 µg/kg bw in clinical trials) than that used to improve hemoglobin (Hb) levels in anemia patients (~0.8 µg/kg bw) [29]. Therefore, rhuEPO^M^ or any of its derivatives at high doses can increase RBC levels and pose a thrombosis risk if they possess hematopoietic activity. We confirmed a lack of erythropoietic activity in asialo-rhuEPO^P^ by repeated IV injection (twice a week for 5 weeks) in female BALB/c mice at a dose of 44 µg/kg bw (neuroprotective dose), which is 55 times higher than the dose of rhuEPO^M^ typically used to stimulate RBC production. At this high dose, asialo-rhuEPO^P^ showed no increase in Hb concentrations, while rhuEPO^M^ significantly increased Hb concentrations as early as a week after two injections [41]. Consistent with this observation, at the end of the 5-week period, the RBC count in mice receiving asialo-rhuEPO^P^ was 7.8 × 10^6^/mm^3^, similar to the saline group (7.5 × 10^6^/mm^3^), while that in mice administered rhuEPO^M^ was 11 × 10^6^/mm^3^, corresponding to a ~47% increase [41]. These results confirmed that asialo-rhuEPO^P^ is also non-erythropoietic, like asialo-rhuEPO^E^.

Concerning the plant-specific sugars on asialo-rhuEPO^P^, there is an ongoing debate whether plant-specific sugars on therapeutic glycoproteins are immunogenic [147,148,149,150]. The immunogenicity of plant-specific sugars on asialo-rhuEPO^P^ was investigated by immunizing BALB/c mice with 44 µg/kg bw (neuroprotective dose) and 88 µg/kg bw protein, along with the same doses of rhuEPO^M^ as a negative control and horse radish peroxidase as a positive control. We detected no antibodies against plant-specific sugars in the sera of mice immunized with asialo-rhuEPO^P^, indicating that it is non-immunogenic even at high doses [41]. These results suggested that asialo-rhuEPO^P^ is safe for use in clinical practice.

### 3.4. Neuroprotective Effects of Asialo-rhuEPO

Asialo-rhuEPO is a nonerythropoietic EPO derivative that has been proven to have neuroprotective functions in various studies (Table 3) [35,39,41,133]. Erbayraktar et al. [35] demonstrated that asialo-rhuEPO^E^ is non-erythropoietic and neuroprotective in animal models of cerebral ischemia, spinal cord compression, and sciatic nerve crush. It has also been shown to attenuate neuronal cell death in the hippocampal CA1 region after transient forebrain ischemia [39]. In addition, asialo-rhuEPO^E^ was reported to improve motor behavior and reduce motoneuron loss in the cervical spinal cord of wobbler mice, an animal model of amyotrophic lateral sclerosis, without affecting hematocrit values [133]. Despite its non-erythropoietic nature and excellent neuroprotective effects, no clinical trials have been conducted for ischemic stroke treatment or for other organ injury treatment, likely due to its limited availability and high cost. To circumvent this problem, we produced asialo-rhuEPO^P^ in tobacco plants. In the following sections, we describe the neuroprotective properties of asialo-rhuEPO^P^ and discuss its potential as a multimodal drug for ischemic stroke treatment.

#### 3.4.1. In Vitro and In Vivo Neuroprotective Effects of Asialo-rhuEPO^P^

We evaluated the in vitro neuroprotective effect of asialo-rhuEPO^P^ by studying its ability to protect neuronal-like cells (N2A) against staurosporine (STS)-induced apoptosis. Our results showed that simultaneous treatment of N2A cells with 1 µM STS and 20 IU/mL asialo-rhuEPO^P^ or rhuEPO^M^ (a positive control) resulted in lower cytotoxicity (47% and 66%, respectively) while treatment with 1 µM STS alone caused 84% cytotoxicity [136]. These results suggest that asialo-rhuEPO^P^ is not only neuroprotective but also more effective (~2-fold) than rhuEPO^M^ in vitro.

The in vivo neuroprotective effect of asialo-rhuEPO^P^ was evaluated using a mouse model of I/R injury and compared with rhuEPO^M^. We used a dose of 44 µg/kg bw because asialo-rhuEPO^E^ has been found to be neuroprotective at this dose in a mouse model of I/R injury [35]. Following IV administration of asialo-rhuEPO^P^ (rhuEPO^M^ as well) immediately at the restoration of blood flow after 1 h occlusion, we observed a significant decrease in neurological deficits (from 3.1 in I/R–saline group to 1.8–1.9 in asialo-rhuEPO^P^ and rhuEPO^M^ groups), cerebral infarction volume (from 33.0% in I/R–saline group to 15.0% in asialo-rhuEPO^P^ and rhuEPO^M^ groups), and edema volume (from ~30% in I/R–saline group to 14% in asialo-rhuEPO^P^ and rhuEPO^M^ groups) [41]. Consistent with these observations, HE and Nissl staining of brain sections showed lesser cellular damage and higher neuron density in the asialo-rhuEPO^P^-treated group than in the I/R–saline group. Immunostaining for NeuN, a marker commonly used to assess the functional state of neurons, also revealed a higher number of NeuN-positive cells in the asialo-rhuEPO^P^-treated group than that in the I/R–saline group [41]. Our results demonstrated that asialo-rhuEPO^P^ is neuroprotective and equipotent to rhuEPO^M^ in reducing brain damage induced by I/R injury.

#### 3.4.2. Neuroprotective Mechanism of Asialo-rhuEPO^P^

Asialo-rhuEPO^E^ has been shown to exert excellent neuroprotection against cerebral I/R injury [35,36,38,39,40,133], but its neuroprotective mechanism has not been dissected yet. Regarding asialo-rhuEPO^P^, our studies suggested that it protects the brain from I/R injury by restoring mitochondria fusion–fission-related proteins, preventing I/R injury-induced mitophagy and autophagy markers, and inhibiting apoptosis [41]. Mitochondria are both a source and target of I/R injury and cell death, and their dysfunction occurring via fission and fusion imbalance is considered one of the hallmarks of I/R-induced neuronal cell death [151,152]. Fission regulates the amounts of mitochondria and removes damaged mitochondria, whereas fusion maintains normal mitochondrial activity by complementing damaged mitochondrial contents with the components of healthy mitochondria [152,153]. I/R injury and other cerebral insults promote fission, leading to disturbed mitochondrial dynamics and compromised mitochondrial functions, thereby promoting the release of pro-apoptotic factors, such as cyt *c* [151,152,154]. Our western blotting and immunofluorescence studies showed that the induced fission-related proteins dynamin-related protein 1 (p-Drp1) and Drp1 receptor fission 1 protein (Fis1) in the brain of the I/R-saline group were significantly restored in both asialo-rhuEPO^P^- and rhuEPO^M^-treated groups [41]. Similarly, the fusion-related proteins mitofusin 1 and 2 (Mfn1 and Mfn2) and optic atrophy protein 1 (OPA1), whose levels were reduced in the I/R-saline group, were significantly restored in asialo-rhuEPO^P^ and rhuEPO^M^ groups [41]. These results suggested that, like rhuEPO^M^, asialo-rhuEPO^P^ can maintain mitochondrial fission–fusion balance under I/R conditions.

Our results also showed that asialo-rhuEPO^P^ treatment and rhuEPO^M^ as well can regulate mitophagy-related markers. Mitochondrial fission is followed by mitophagy to remove damaged organelles [155,156]. In mammalian cells, the PINK1 (PTEN-induced putative kinase protein 1) and parkin (an E3 ubiquitin ligase PARK2) cooperatively sense cellular stress and mediate the removal of damaged mitochondria [155,156]. Our Western blotting results showed that asialo-rhuEPO^P^ (also rhuEPO^M^) treatment was able to restore both PINK1 and PARK2 levels that were elevated by I/R injury [41], consistent with lower mitochondrial fission anticipated from the restoration of fission-related proteins in EPO-treated groups. In addition to mitophagy, our Western blotting and immunofluorescence results of general autophagy markers (LC3B, p62, and Beclin1) revealed that their increased levels in the I/R–saline group were reinstated back in asialo-rhuEPO^P^ and rhuEPO^M^ groups to the levels similar to the sham group [41]. Furthermore, investigation of apoptotic markers and TUNEL staining revealed that asialo-rhuEPO^P^-treatment (also rhuEPO^M^) significantly attenuated I/R-induced Bax/Bcl2 ratio, cyt *c* release, and caspase 3 caspase cleavage, and reduced TUNEL-positive cells by 50% compared to the I/R–saline control, indicating that both asialo-rhuEPO^P^ and rhuEPO^M^ have similar suppressive effects on I/R injury-induced apoptosis [41]. Together, these results suggested that asialo-rhuEPO^P^- and rhuEPO^M^-treatment can restore I/R injury-affected mitochondrial fission–fusion-related proteins and mitophagy- and autophagy-related markers, leading to anti-apoptotic effects and cell survival.

Regarding signaling pathways responsible for displaying the neuroprotective effect of asialo-rhuEPO^P^, our Western blotting results showed a significant increase in phosphorylation of STAT5, PI3K, AKT, and ERK1/2 in asialo-rhuEPO^P^- and rhuEPO^M^-treated groups compared with the I/R–saline group [41]. These results suggest that, like rhuEPO^M^, the neuroprotective effects of asialo-rhuEPO^P^ are mediated through the activation of STAT5, PI3K/AKT, and MAPK/ERK1/2 signaling pathways as reported previously by others for rhuEPO^M^ [71,101,102,103,104]. These findings also indicate that asialo-rhuEPO^P^, like rhuEPO^M^, exhibits pleiotropic effects through these pathways. However, at this time, it remains unknown what the relative contribution of each of these asialo-rhuEPO^P^-induced signaling pathways is to the regulation of mitochondrial fission–fusion, mitophagy, autophagy, and apoptosis to display neuroprotective effects. In addition, the receptor responsible for transducing asialo-rhuEPO^P^ tissue-protective signal remains to be identified.

## 4. Future Directions

Since the preclinical results of rhuEPO^M^ to protect against I/R injuries in the brain and other organs could not be consistently observed in clinical trials, more precautions should be taken to perform a clinical trial for ischemic stroke treatment with asialo-rhuEPO, regardless of whether asialo-rhuEPO^E^ or asialo-rhuEPO^P^ is used. Several preclinical experiments may still be required to obtain more information in order to determine whether it is worth carrying out an ischemic stroke clinical study with asialo-rhuEPO.

Firstly, the neuroprotective effects of asialo-rhuEPO should be investigated in an aged-stroke mouse model, as age is the most important risk factor for cerebral ischemia [157,158,159,160]. Most studies on cerebral I/R injury and its therapies were performed in young and healthy animals [10,160]. These young and healthy animals used in preclinical studies are dramatically different from patients recruited into clinical trials who are usually of advanced age with different health conditions [160,161,162]. Furthermore, elderly patients have a higher burden of surgical risk factors with reduced functional capacity and increased comorbidities compared to younger patients [161,163]. The aged brain often displays a compromised ability to resolve stroke-mediated inflammation and shows poor functional recovery compared to the young brain [158,160]. Ischemic stroke experiments with aged rats have also shown significant differences in neuroinflammation, accelerated apoptosis, and precipitous infarct development compared to young and healthy animals after an ischemic insult [164]. Hence, preclinical studies with young animal models might not represent aged acute IS patients well [162,165]. It is essential to study neuroprotective effects of the asialo-rhuEPO in aged animal models, which more closely mimics the clinical situation.

Secondly, it is necessary to investigate the dosage of asialo-rhuEPO to determine an optimal dose and subsequent therapeutic time window. High doses and repeated administration of both asialo-rhuEPO^E^ and asialo-rhuEPO^P^ revealed no increase in either RBC counts or Hb levels [35,41]. These results are good indicators that asialo-rhuEPO is indeed non-erythropoietic, leading us to believe that it should be free of HAASEs. The asialo-rhuEPO dose commonly used in animal experiments is ~5000 IU/kg bw [35,41], which was based on the previously optimal dose found in studies on rhuEPO^M^ [18,124]. Further studies with different doses of asialo-rhuEPO in stroke animal models could allow us to determine whether its dose can be increased further to bring better or stable neuroprotective effects. A previous study also revealed that the best administration time is up to 3 h after ischemia with a leaky BBB [166]. It will be also interesting to know under the optimal dose whether the administration time window of asialo-rhuEPO can be extended or not.

Thirdly, it is also important to know whether asialo-rhuEPO without hematopoietic activity will bring out better long-term neuroprotective functions to minimize I/R-induced brain injury than rhuEPO^M^ with hematopoietic activity. Currently, most neuroprotective effects of asialo-rhuEPO treatment were observed in short-term studies with the doses optimized from rhuEPO^M^ studies. The short-term neuroprotective effects of asialo-rhuEPO were found to be similar to those observed in rhuEPO^M^ treatment [35,41]. Based on their different hematopoietic properties, asialo-rhuEPO^P^ free of HAASEs might have better long-term neuroprotective functions than rhuEPO^M^ when they are used to treat I/R-induced brain injury. Therefore, investigating its long-term rather than short-term effects, especially in aged mice, would be more meaningful before moving asialo-rhuEPO to clinical tests.

Lastly, the tissue-protective receptor for EPO remains elusive [6,167]. RhuEPO^M^ and asialo-rhuEPO with different terminal sugars and protein charges may use distinct receptors and certain unique mechanisms to attenuate I/R injury. To understand whether two different activities (erythropoiesis and tissue protection) of EPO use different receptors and to identify any alternative tissue-protective EPO receptor have basic and practical significance, which will lay a solid foundation to exploit EPO derivatives like asialo-rhuEPO as potential tissue-protective drugs. Hence, further investigation of whether the erythropoietic and tissue-protective activities of EPO are mediated through the same receptor or different receptor(s) is crucial for future applications. Asialo-rhuEPO displays only a cytoprotective function, and could be an ideal EPO derivative to be used to identify and study EPO tissue-protective receptor(s).

## Figures and Tables

**Table 1:** General information on EPO, rhuEPO^M^, asialo-rhuEPO^E^, and asialo-rhuEPO^P^.

Type	Source	Terminal Sugar	Properties	Activities
Endogenous EPO	The major amount is produced by the kidneys in adults and small amounts by other organs	Sialic acid	Molecular weight: ~30.4 kDa; pI: 3.92–4.42; half-life in the circulation system: ~5 h	Erythropoiesis; tissue protection
RhuEPO^M^	Overexpressing human EPO gene in mammalian cells	Sialic acid	Molecular weight: 26–36 kDa; pI: 4.42–5.11; half-life in the circulation system: 4–8 h	Erythropoiesis; tissue protection
Asialo-rhuEPO^E^	Enzymatic removal of sialic acids from rhuEPO^M^	β1,4-Galactose	Molecular weight: undetermined; pI: ~8.5; half-life in the circulation system: 2–3 min	Tissue protection
Asialo-rhuEPO^P^	Overexpressing human EPO and GalT genes in plants	β1,4-Galactose	Molecular weight: 28–30 kDa; pI: ~8.75; half-life in the circulation system: undetermined.	Tissue protection

**Table 2:** Clinical trials of rhuEPO^M^ treatment for acute ischemic stroke patients.

Number of Patients	Dosage	Route	Time of Evaluation	Results	Ref.
rhuEPO^M^ (*n* = 21); placebo (*n* = 19)	99,000 IU (33,000 IU each within 8 h of symptom onset, and followed up at 24 and 48 h)	IV	30 days	Reduced infarct size, improved recovery of neurocognitive function and the neurological deficit, and ameliorated stroke-related disability at 30 days	[31]
rhuEPO^M^ (*n* = 37); placebo (*n* = 43)	56,000 IU (16,000 IU as a bolus dose followed by 8000 IU each at 12 h intervals for remaining 5 doses)	IV	28 days	Effective reduction of ischemic stroke complication	[33]
rhuEPO^M^ and placebo each (*n* = 71)	10,000 IU (5000 IU each at 48 and 72 h after stroke)	SC	90 days and 5 years	Reduced the scale of Barthel index but did not affect long-term recurrent stroke and mortality; significantly improved long-term neurological outcomes	[34]
rhuEPO^M^ (*n* = 256); placebo (*n* = 266)	120,000 IU (40,000 IU each within 6 h of symptom onset, and followed up at 24 and 48 h)	IV	90 days	The treatment of rhuEPO^M^ or combined with rtPA did not show any improvement in clinical outcomes but had a higher overall death rate	[32] *

*: Some patients from both rhuEPO^M^ (*n* = 166) and placebo (*n* = 165) groups were treated with rtPA together.

**Table 3:** In vitro and in vivo neuroprotective effects of asialo-rhuEPO.

Author/Year	Cell Line/Animal	Treatment/Model	Outcome
Erbayraktar et al. [35]/2003	PC-12 cells	Nerve growth factor (NGF) absence-triggered cell death	34% protection
Erbayraktar et al. [35]/2003	P-19 cells	Hypoxia for 15 h	43% protection
Mennini et al. [133]/2006	Motoneuron culture	Kainate-induced cell death	Increased survival rate by 57%
Ishii et al. [40]/2012	PC-12 cells	Nerve growth factor (NGF) absence-triggered cell death	No observed protection
Kittur et al. [136]/2013	N2A cells	Staurosporine-induced cell death	44% protection
Erbayraktar et al. [35]/2003	Sprague Dawley male rats	MCAO model	Reduced infarct volume by ~50%
Erbayraktar et al. [35]/2003	Sprague Dawley male rats	Spinal cord compression	Restricted injury with better neuron survival and motor score
Erbayraktar et al. [35]/2003	Sprague Dawley male rats	Sciatic nerve crush model	Reduced functional loss and improved motor testing score
Wang et al. [36]/2004	Wistar rat pups (7 days old)	Hypoxia–ischemia model	Reduced infarct volume by 52%
Grasso et al. [37]/2006	Sprague Dawley rats	Spinal cord compression	Significantly recovered affected motor function
Mennini et al. [133]/2006	Homozygous wobbler mice	Amyotrophic lateral sclerosis model carrying a mutation of Vps54 gene	Improved motor behavior and reduced inflammation
Price et al. [38]/2010	Sprague Dawley male rats	MCAO model	Significantly reduced infarct volume with reduced cell death
Yamashita et al. [39]/2010	Mongolian male gerbils	Occlusion of the common carotid arteries	Improved learning and memory function with better neuron survival
Ishii et al. [40]/2012	Wistar male rats	MCAO model	Significantly reduced cerebral I/R injury
He et al. [41]/2022	BALB/c male mice	MCAO model	Significant decreased neurological deficits, infarction volume, and edema volume with better neuron survival

## Data Availability

Data sharing not applicable.
